# Cumulative inactivated vaccine exposure and allergy development among children: a birth cohort from Japan

**DOI:** 10.1186/s12199-020-00864-7

**Published:** 2020-07-07

**Authors:** Kiwako Yamamoto-Hanada, Kyongsun Pak, Mayako Saito-Abe, Limin Yang, Miori Sato, Hidetoshi Mezawa, Hatoko Sasaki, Minaho Nishizato, Mizuho Konishi, Kazue Ishitsuka, Kenji Matsumoto, Hirohisa Saito, Yukihiro Ohya, Japan Environment and Children’s Study (JECS) Group, Shin Yamazaki, Yukihiro Ohya, Reiko Kishi, Nobuo Yaegashi, Koichi Hashimoto, Chisato Mori, Shuichi Ito, Zentaro Yamagata, Hidekuni Inadera, Michihiro Kamijima, Takeo Nakayama, Hiroyasu Iso, Masayuki Shima, Youichi Kurozawa, Narufumi Suganuma, Koichi Kusuhara, Takahiko Katoh

**Affiliations:** 1 Allergy Center, National Center for Child Health and Development, Tokyo, Japan; 2 Medical Support Center for the Japan Environment and Children’s Study, National Research Institute for Child Health and Development, Tokyo, Japan; 3 Division of Biostatistics, Department of Data Management, Center for Clinical Research and Development, National Center for Child Health and Development, Tokyo, Japan; 4 Department of Allergy and Clinical Immunology, National Research Institute for Child Health and Development, Tokyo, Japan

**Keywords:** Adjuvant, Asthma, Eczema, Wheeze, Inactivated vaccine

## Abstract

**Background:**

Adjuvants used in inactivated vaccines often upregulate type 2 immunity, which is dominant in allergic diseases. We hypothesised that cumulative adjuvant exposure in infancy may influence the development of allergies later in life by changing the balance of type 1/type 2 immunity. We examined the relationship between immunisation with different vaccine types and later allergic disease development.

**Methods:**

We obtained information regarding vaccinations and allergic diseases through questionnaires that were used in The Japan Environment and Children’s Study (JECS), which is a nationwide, multicentre, prospective birth cohort study that included 103,099 pregnant women and their children. We examined potential associations between the initial vaccination before 6 months of age and symptoms related to allergies at 12 months of age.

**Results:**

Our statistical analyses included 56,277 children. Physician-diagnosed asthma was associated with receiving three (aOR 1.395, 95% CI 1.028–1.893) or four to five different inactivated vaccines (aOR 1.544, 95% CI 1.149–2.075), compared with children who received only one inactivated vaccine. Similar results were found for two questionnaire-based symptoms, i.e. wheeze (aOR 1.238, 95% CI 1.094–1.401; three vaccines vs. a single vaccine) and eczema (aOR 1.144, 95% CI 1.007–1.299; four or five vaccines vs. a single vaccine).

**Conclusions:**

Our results, which should be cautiously interpreted, suggest that the prevalence of asthma, wheeze and eczema among children at 12 months of age might be related to the amount of inactivated vaccine exposure before 6 months of age. Future work should assess if this association is due to cumulative adjuvant exposure. Despite this possible association, we strongly support the global vaccination strategy and recommend that immunisations continue.

**Trial registration:**

UMIN000030786.

## Introduction

Universal childhood vaccination is very important and plays a role in saving the lives of children. Vaccines stimulate the immune system to protect an individual against future infections. There is clear evidence that immunisation by the administration of vaccines is effective for controlling and preventing various life-threatening infectious diseases. Unlike live vaccines, inactivated vaccines often require adjuvants to generate an immune response because the immunogenicity of the inactivated vaccines is generally lower and their effective period is shorter compared with live vaccines [1]. Aluminium is the most commonly used adjuvant in inactivated vaccines [2]. However, aluminium adjuvants are known to induce type 2 immune responses. Clinical trials have tested all adjuvants that are currently used in vaccines for safety and effectiveness, and these adjuvants have been used safely in clinical practice for many years [3]. In a mouse model, it is well-known that IgE production is enhanced by presenting antigens with aluminium adjuvants [4]. For basic science research, it is common to enhance allergic reactions in animal models using aluminium adjuvants; however, the activated pathways and effects stimulated by aluminium adjuvants in people may differ from these models because findings from animals do not always translate into humans.

The association of inactivated vaccines with allergic diseases remains controversial. In support of this idea, Kiraly et al. showed that delayed diphtheria, tetanus and acellular pertussis (DTaP) vaccination, defined as giving the first dose after 90 days of age (1 month later than usual), was associated with reduced eczema and use of eczema medication in Australia [5]. Schlaud et al. reported that children in Germany who completed TDPHiHeP immunisation (tetanus, diphtheria, poliomyelitis, *Haemophilus influenza* type b; Hib, hepatitis B and pertussis) at the end of the first year of life had a lower risk of subsequently developing hay fever [6]. In contrast, two different studies reported that routine immunisation against pertussis in infancy did not increase the risk of asthma or wheeze [7, 8].

Unlike inactivated vaccines whose adjuvants may promote type 2 immunity, the bacillus Calmette–Guérin (BCG) vaccine promotes the development of type 1 helper T (Th1) and IL-17-producing helper T (Th17) cells [9]. Although some studies have suggested that BCG vaccination is protective against the development of allergic diseases, a systematic review and meta-analysis concluded that BCG vaccination was not associated with development of eczema, rhinoconjunctivitis, allergic sensitization, or allergic diseases in general [10]. Notably, that review included only retrospective cohort studies, cross-sectional studies and a small RCT, and did not include any prospective cohort studies; therefore, the evidence level of the review does not seem very high. Further study is needed to confirm or exclude the presence of an association between BCG vaccination and allergic diseases.

Allergies, such as pollinosis, are a serious health concern in Japan. The prevalence of asthma and eczema among Japanese children is 13.3% and 24.5%, respectively [11]. According to a nationwide birth cohort study, about 36% of pregnant women in Japan have a history of pollinosis and 55.6% are IgE-sensitised to Japanese cedar [12]. Given the relatively high prevalence of allergies in Japan, it is important to investigate potential triggers and preventions for the development of this condition.

We hypothesised that adjuvant exposure through early immunisation (before 6 months of age) could have a crucial influence on the development of allergic diseases. This study addressed three research questions (RQs). Our first RQ (RQ1) was whether babies who receive their first inoculations with inactivated vaccines, and are consequently exposed to adjuvant, later develop more allergic diseases than those first inoculated with live vaccines (no adjuvant required) (RQ1). Our second RQ (RQ2) was whether simultaneous inoculation with a greater number of inactivated vaccines (as a proxy for adjuvant exposure) leads to a proportionately greater risk of subsequently developing allergic diseases. Finally, our third RQ (RQ3) was whether BCG vaccination reduces the risk of allergic diseases. To answer these RQs, we examined the relationship between initial (i.e. the first time in life; before 6 months of age) vaccine type exposure and later allergic disease development in the general population of Japan.

## Methods

### Study population

Although it is not a clinical trial, the Japan Environment and Children’s Study (JECS) is registered with the University Hospital Medical Information Network Clinical Trials Registry, number UMIN000030786. The JECS is a nationwide, multicentre, prospective birth cohort study conducted by the Ministry of Environment of Japan, and its protocol and major hypotheses have been published as a protocol paper [12–15]. A total of 103,099 pregnant women were recruited from the general population of Japan for the JECS at 15 research centres covering a wide geographical area of Japan, from the north of the country (Hokkaido) to the south (Okinawa), from April 2011 to March 2014. The pregnancies of the recruited women resulted in 104,065 newborns. The present study was conducted as a part of the JECS, and we performed statistical analyses using the released data sets (jecs-ag-20160424 and jecs-an-20180131).

### Ethics statement

The JECS protocol was reviewed and approved by the Ministry of the Environment’s Institutional Review Board on Epidemiological Studies and by the ethics committees of all participating institutions. Written informed consent was obtained from all participants (i.e. pregnant women or other caregivers). The JECS was conducted in accordance with the Helsinki Declaration and other nationally valid regulations and guidelines.

### Questionnaires and medical records

Written questionnaires were mailed to caregivers during pregnancy and when each child was 6 months and 12 months of age. Caregivers answered questions regarding the mothers and infants during pregnancy and at ages 6 and 12 months. In addition, we compiled birth and neonatal information, including the obstetric outcomes, from the medical records provided by each participant’s institution.

### Outcomes, exposures and covariates

The outcome definitions for this study are defined in the file S1. Prevalence of allergic diseases (asthma, atopic dermatitis, food allergy and any other allergies) was assessed based on a parent-reported doctor’s diagnosis, obtained from participant questionnaires when the children were 12 months old. Wheeze and eczema symptoms were evaluated at age 12 months based on a questionnaire from the International Study of Asthma and Allergies in Childhood (ISAAC) [16]. Vaccination history was obtained from the questionnaires completed at age 6 months. Caregivers of the children checked the types of initial vaccination on the questionnaire. Diphtheria-tetanus-pertussis vaccine (DPT) was defined as a single vaccine. The types of polio vaccination administered in Japan differed by the date [17, 18]. Live polio vaccine was administered until August, 2012. Subsequently, inactivated polio virus vaccine became available in September, 2012 and DPT-inactivated polio vaccine (IPV) in November, 2012. Therefore, based on the birth date, we categorised polio vaccines into either live or inactivated vaccine. The DPT-IPV was counted as another inactivated vaccine type, i.e. counted as two types.

### Target populations for statistical analysis

For RQ1, we included the subset of infants whose initial vaccination was with inactivated vaccine(s) only and those whose initial vaccination was with live vaccine(s) only. For RQ2, we included infants whose first vaccination was with inactivated vaccines only. For RQ3, we included infants whose initial vaccination was with BCG only and those whose initial vaccination was with rotavirus only.

### Statistical analyses

After excluding multiple births and cases with missing variables, we performed statistical analyses of the remaining data (Figure S1). Based on clinical experience and a review of past reports, we assessed the following factors as possible confounders in the regression analyses: maternal age, primiparity, maternal allergy history, smoking during pregnancy, pet ownership during pregnancy, sex of the child, feeding at 6 months, nursery school attendance, maternal education level, annual family income and place of recruitment. We checked the collinearity of the preliminarily selected confounders and included all the available variables in the model without performing variable selection. For each outcome variable of allergic features and for the exposure variables in each RQ, we conducted logistic regression analyses in which all potential confounders were included. We evaluated the adjusted odds ratios (aORs) and odds ratios (ORs) as measures of association and calculated 95% confidence intervals (CIs) and *p* values. The adjusted *p* values were calculated by the Holm method. For RQ2, we performed a multiplicity adjustment by Dunnett’s method, with the number of inactivated vaccines set to 1 as a reference. For each model, we checked multicollinearity with the variance inflation factor, using a cut-off value of 5. In addition, as sensitivity analyses for RQ1 (*n* = 56,277) and RQ3 (*n* = 8012), we estimated aORs by implementing a multivariate imputation (MI) using a chained equations (MICE) algorithm with 20 iterations and using the R package MICE, version 3.3.0 [19]. We did not perform imputation for RQ2 because we were able to confirm the same tendency as RQ1 based on the MI results. All available confounders were used in the imputation equations.

This was an exploratory study to estimate the magnitude of associations between allergy and vaccination; therefore, we did not define the significance level of *p* values. We also performed Bayesian multivariable logistic regression using the R package rstan, version 2.17.3 [20], to estimate the posterior distribution of the aORs. We applied multiple statistical analyses, i.e., both Frequentist and Bayesian approaches, to evaluate the association measures from multiple perspectives. We set vague priors for all coefficients using a normal distribution with a mean of 0 and a variance of 10,000, and generated a posterior distribution sample by Markov chain Monte Carlo methods (No-U-Turn sampler) with 11,000 iterations, four chains, four thinning intervals and 1000 warm-up times. We assessed the posterior distribution of aORs with 25th, 50th, and 75th percentiles, 95% credible intervals (CrIs) and the probabilities of aOR > 1.0, > 1.5 and > 2.0. All statistical analyses were performed using R statistical software, version 3.5.1 (The R Foundation, Vienna, Austria) [21].

## Results

The prevalence of wheeze, eczema and physician-diagnosed asthma at 1 year of age for the entire cohort of study participants was 19.9%, 18.4–18.7% and 2.5–2.6%, respectively (see Table S1). The prevalence of allergic features by each RQ is described in Tables S5, S6 and S7.

### RQ1: initial immunisation with inactivated vaccines only vs. with live vaccines only

To answer the question of whether or not initial inoculation with inactivated vaccines could lead to more frequent development of allergic diseases compared with initial inoculation using live vaccines, we compared subjects whose first vaccination was with inactivated vaccine(s) only to those whose first vaccination was with live vaccine(s) only. Regarding their initial vaccination, 8165 children received live vaccine(s) only and 48,112 children received inactivated vaccine(s) only (Table S2). The children who received inactivated vaccine(s) only did not show any associations with allergic features at 1 year of age compared with those who received live vaccine(s) only (Table 1). Based on the results of a Bayesian multivariable logistic regression, the posterior probabilities of aOR > 1.0 were 0.642 for food allergy, 0.488 for asthma and 0.603 for eczema (none of these are significant; Table 2). The results for complete case and MI case data did not differ.

**Table 1:** Results of univariable and multivariable logistic regression for allergic features associated with initial inactivated vaccine compared with initial live vaccine only, in the JECS birth cohort (RQ1)

Allergy features at 1 year old	Univariable				Multivariable for complete cases				Multivariable for multiple imputed data			
	OR	Lower 95% CI	Upper 95% CI	*p* value	aOR	Lower 95% CI	Upper 95% CI	*p* value	aOR	Lower 95% CI	Upper 95% CI	*p* value
Allergic disease	0.984	0.935	1.035	0.521	0.994	0.940	1.051	0.832	0.985	0.935	1.037	0.553
Atopic dermatitis	0.909	0.813	1.016	0.093	0.943	0.834	1.065	0.341	0.914	0.816	1.023	0.118
Food allergy	1.008	0.947	1.074	0.805	1.013	0.946	1.084	0.720	1.011	0.948	1.078	0.736
Asthma	1.027	0.885	1.192	0.724	0.989	0.840	1.164	0.894	1.033	0.887	1.203	0.679
Wheeze	1.001	0.944	1.062	0.963	0.980	0.917	1.047	0.550	0.991	0.932	1.054	0.776
Eczema	0.978	0.921	1.039	0.474	1.009	0.944	1.078	0.797	0.992	0.933	1.054	0.785

*OR* odds ratio, *aOR* adjusted odds ratio, *CI* confidence interval

aOR and 95% CIs were estimated with potential confounders including maternal age, primiparity, maternal allergy history, smoking during pregnancy, pet ownership during pregnancy, sex of child, feeding at 6 months, nursery school attendance, maternal education level, annual family income and place of recruitment

**Table 2:** Results of Bayesian multivariable logistic regression for allergic symptoms using vaccine types and covariates for complete cases (RQ1)

Outcome	aOR P25	aOR P50	aOR P75	95% CrI lower	95% CrI upper	Pr (aOR >1.0)	Pr (aOR >1.5)	Pr (aOR >2.0)
Allergic disease	0.976	0.995	1.013	0.941	1.051	0.420	<0.0001	<0.0001
Atopic dermatitis	0.905	0.943	0.983	0.834	1.064	0.177	<0.0001	<0.0001
Food Allergy	0.989	1.013	1.037	0.947	1.084	0.642	<0.0001	<0.0001
Asthma	0.936	0.990	1.046	0.843	1.167	0.448	<0.0001	<0.0001
Wheeze	0.958	0.980	1.003	0.917	1.047	0.275	<0.0001	<0.0001
Eczema	0.986	1.009	1.032	0.947	1.078	0.603	<0.0001	<0.0001

Parameters: iterations = 11000, chains = 4, thinning = 4, warm-ups = 1000

*aOR* adjusted odds ratio, *CrI* credible interval, *Pr* probability, *P25* 25th percentile, *P50* 50th percentile, *P75* 75th percentile

aORs were estimated with potential confounders including maternal age, primiparity, maternal allergy history, smoking during pregnancy, pet ownership during pregnancy, sex of child, feeding at 6 months, nursery school attendance, maternal education level, annual family income and place of recruitment

### RQ2: Initial immunisation with multiple inactivated vaccines

RQ2 aimed to examine whether there was a dose-dependent relationship between receiving multiple inactivated vaccines simultaneously at the time of first vaccination and a greater risk of later development of allergic diseases. The percentages of infants who received only one type (including the triple vaccine DPT), two, three and four to five different types of inactivated vaccines were 21.2%, 64.0%, 7.7% and 7.2%, respectively (Table S3). Physician-diagnosed asthma was associated with receiving three types of inactivated vaccine (aOR 1.395, 95% CI 1.028–1.893) and four or five types of inactivated vaccine (aOR 1.544, 95% CI 1.149–2.075) (Table 3). ISAAC-based wheeze symptom was associated with receiving three different inactivated vaccines at the initial immunisation (aOR 1.238, 95% CI 1.094–1.401), and ISAAC-based eczema was associated with receiving four or five different inactivated vaccines at the initial immunisation (aOR 1.144, 95% CI 1.007–1.299). The posterior probabilities of aORs were also increased for asthma, wheeze and eczema in Bayesian multivariable logistic regression analyses (Table 4). In particular, regarding the association of asthma with the administration of four or five types of inactivated vaccine at the initial immunisation, the posterior probability of aOR > 1.0 was 1.000, that of aOR > 1.5 was 0.581 and that of aOR > 2.0 was 0.012 (95% CrI 1.198–1.967). As for eczema, the posterior probability of aOR > 1.0 was 0.992 (95% CrI 1.027–1.269).

**Table 3:** Results of univariable and multivariable logistic regression for allergic features associated with number of initial inactivated vaccines received, in the JECS birth cohort (RQ2)

Allergy features at 1 year old	Number of inactivated vaccines	Univariable				Multivariable for complete cases			
		OR	Lower 95% CI	Upper 95% CI	*p* value	aOR	Lower 95% CI	Upper 95% CI	*p* value
Allergic disease	2 vs. 1	1.012	0.953	1.072	0.932	0.993	0.932	1.059	0.710
	3 vs. 1	1.031	0.934	1.137	0.932	1.044	0.938	1.162	0.686
	4 or 5 vs. 1	1.075	0.973	1.188	0.263	1.098	0.985	1.223	0.125
Atopic dermatitis	2 vs. 1	0.957	0.837	1.093	0.683	0.947	0.820	1.094	1.000
	3 vs. 1	1.116	0.900	1.384	0.683	1.076	0.851	1.360	1.000
	4 or 5 vs. 1	1.113	0.893	1.386	0.683	1.095	0.866	1.385	1.000
Food allergy	2 vs. 1	0.939	0.875	1.009	0.075	0.959	0.888	1.035	0.579
	3 vs. 1	0.886	0.784	1.002	0.059	0.965	0.847	1.101	1.000
	4 or 5 vs. 1	0.914	0.807	1.036	0.089	0.963	0.842	1.101	1.000
Asthma	2 vs. 1	1.278	1.063	1.538	0.002	1.201	0.981	1.470	0.034
	3 vs. 1	**1.662**	**1.268**	**2.179**	< **0.0001**	**1.395**	**1.028**	**1.893**	**0.021**
	4 or 5 vs. 1	**1.800**	**1.375**	**2.356**	< **0.0001**	**1.544**	**1.149**	**2.075**	**0.002**
Wheeze	2 vs. 1	1.035	0.966	1.109	0.240	0.990	0.916	1.069	0.752
	3 vs. 1	**1.286**	**1.152**	**1.435**	< **0.0001**	**1.238**	**1.094**	**1.401**	< **0.0001**
	4 or 5 vs. 1	**1.248**	**1.114**	**1.397**	< **0.0001**	1.130	0.995	1.283	0.046
Eczema	2 vs. 1	1.021	0.952	1.096	0.485	1.006	0.932	1.086	0.851
	3 vs. 1	1.086	0.966	1.220	0.191	1.113	0.980	1.261	0.095
	4 or 5 vs. 1	1.116	0.992	1.257	0.084	**1.144**	**1.007**	**1.299**	**0.038**

*OR* odds ratio, *aOR* adjusted odds ratio, *CI* confidence interval

aOR and 95% CIs were estimated with potential confounders including maternal age, primiparity, maternal allergy history, smoking during pregnancy, pet ownership during pregnancy, sex of child, feeding at 6 months, nursery school attendance, maternal education level, annual family income and place of recruitment. The adjusted *p* values were calculated by Holm method. Results of *p* value < 0.05 are shown in bold

**Table 4:** Results of Bayesian multivariable logistic regression for allergic features associated with number of initial inactivated vaccines for complete cases (RQ2)

	Variable: number of vaccines	aOR_P25	aOR_P50	aOR_P75	95% CrI_lower	95% CrI_upper	Pr (aOR > 1.0)	Pr (aOR > 1.5)	Pr (aOR > 2.0)
Allergic disease	2 vs. 1	0.975	0.993	1.011	0.941	1.047	0.397	< 0.0001	< 0.0001
	3 vs. 1	1.012	1.045	1.077	0.956	1.141	0.825	< 0.0001	< 0.0001
	4 or 5 vs. 1	1.063	1.097	1.133	**1.003**	**1.201**	**0.978**	< 0.0001	< 0.0001
Atopic dermatitis	2 vs. 1	0.909	0.947	0.989	0.841	1.067	0.194	< 0.0001	< 0.0001
	3 vs. 1	1.002	1.073	1.147	0.885	1.300	0.758	0.001	< 0.0001
	4 or 5 vs. 1	1.023	1.095	1.170	0.810	1.330	0.815	0.001	< 0.0001
Food allergy	2 vs. 1	0.939	0.959	0.980	0.899	1.020	0.090	< 0.0001	< 0.0001
	3 vs. 1	0.930	0.965	1.002	0.864	1.074	0.263	< 0.0001	< 0.0001
	4 or 5 vs. 1	0.927	0.963	1.000	0.862	1.076	0.250	< 0.0001	< 0.0001
Asthma	2 vs. 1	1.133	1.202	1.275	**1.015**	**1.434**	**0.982**	0.007	< 0.0001
	3 vs. 1	1.279	1.392	1.518	**1.072**	**1.799**	**0.992**	0.281	0.004
	4 or 5 vs. 1	1.413	1.538	1.676	**1.198**	**1.967**	**1.000**	0.581	0.019
Wheeze	2 vs. 1	0.969	0.990	1.012	0.929	1.056	0.377	< 0.0001	< 0.0001
	3 vs. 1	1.195	1.237	1.281	**1.116**	**1.374**	**1.000**	< 0.0001	< 0.0001
	4 or 5 vs. 1	1.089	1.129	1.171	**1.016**	**1.254**	**0.987**	< 0.0001	< 0.0001
Eczema	2 vs. 1	0.985	1.007	1.029	0.945	1.072	0.578	< 0.0001	< 0.0001
	3 vs. 1	1.072	1.112	1.153	0.999	1.234	0.974	< 0.0001	< 0.0001
	4 or 5 vs. 1	1.103	1.144	1.187	**1.027**	**1.269**	**0.992**	< 0.0001	< 0.0001

Parameters: iterations = 11000, chains = 4, thinning = 4, warm-ups = 1000.

*aOR* adjusted odds ratio, *CrI* credible interval, *Pr* probability, *P25* 25th percentile, *P50* 50th percentile, *P75* 75th percentile

Results of 95% CrI > 1.0 and probability > 0.975 are shown in bold

aORs were estimated with potential confounders including maternal age, primiparity, maternal allergy history, smoking during pregnancy, pet ownership during pregnancy, sex of child, feeding at 6 months, nursery school attendance, maternal education level, annual family income and place of recruitment

### RQ3: BCG vaccination vs. rotavirus vaccination

RQ3 aimed to examine whether BCG vaccination as the initial immunisation reduces the risk of subsequent allergic diseases. The statistical analyses for RQ3 included 6093 infants vaccinated with BCG only and 1919 infants immunised with rotavirus vaccine only at their initial immunisations (Table S4). There were no differences in the allergic features between children whose initial vaccination was with BCG only and those whose initial immunisation was with rotavirus vaccine only, in multivariable logistic regression analyses of both complete case data and multiple imputation (MI) data (Table 5). In the analyses using Bayesian multivariable logistic regression, the posterior probability of aOR > 1.0 was 0.841, that of aOR > 1.5 was 0.165 and that of aOR > 2.0 was 0.008 (95% CrI 0.809–1.832) for asthma (Table 6). The results for complete case and MI case data did not differ.

**Table 5:** Results of univariable and multivariable logistic regression for allergic features associated with initial live polio vaccine only compared with initial BCG vaccine only, in the JECS birth cohort (RQ3)

Allergy features at 1 year old	Univariable				Multivariable for complete cases				Multivariable for multiple imputed data			
	OR	Lower 95% CI	Upper 95% CI	*p* value	aOR	Lower 95%CI	Upper 95% CI	*p* value	aOR	Lower 95% CI	Upper 95% CI	*p* value
Allergic disease	1.032	0.924	1.153	0.578	0.970	0.851	1.105	0.648	0.990	0.123	7.966	0.868
Atopic dermatitis	0.872	0.678	1.122	0.287	0.889	0.663	1.192	0.430	0.833	0.087	7.936	0.191
Food allergy	1.150	1.005	1.315	0.042	1.060	0.906	1.242	0.466	1.089	0.132	9.015	0.259
Asthma	0.770	0.543	1.092	0.142	1.234	0.822	1.852	0.311	1.148	0.106	12.486	0.484
Wheeze	0.914	0.802	1.041	0.175	1.068	0.912	1.2525	0.413	1.089	0.132	9.009	0.253
Eczema	0.987	0.865	1.127	0.845	0.887	0.759	1.037	0.131	0.930	0.113	7.674	0.327

*OR* odds ratio, *aOR* adjusted odds ratio, *CI* confidence interval

aOR and 95% CIs were estimated with potential confounders including maternal age, primiparity, maternal allergy history, smoking during pregnancy, pet ownership during pregnancy, sex of child, feeding at 6 months, nursery school attendance, maternal education level, annual family income and place of recruitment

**Table 6:** Results of Bayesian multivariable logistic regression for allergic features associated with initial rotavirus vaccine only compared with initial BCG only, in the JECS birth cohort for complete cases (RQ3)

Outcome	aOR P25	aOR P50	aOR P75	95% CrI lower	95% CrI upper	Pr (aOR > 1.0)	Pr (aOR > 1.5)	Pr (aOR > 2.0)
Allergic disease	0.928	0.971	1.015	0.851	1.105	0.327	< 0.0001	< 0.0001
Atopic dermatitis	0.798	0.884	0.977	0.656	1.181	0.205	< 0.0001	< 0.0001
Food allergy	1.005	1.060	1.119	0.904	1.234	0.770	< 0.0001	< 0.0001
Asthma	1.069	1.230	1.410	0.809	1.823	0.841	0.165	0.008
Wheeze	1.012	1.069	1.129	0.912	1.250	0.795	< 0.0001	< 0.0001
Eczema	0.839	0.887	0.934	0.758	1.036	0.063	< 0.0001	< 0.0001

Parameters: iterations = 11000, chains = 4, thinning = 4, warm-ups = 1000

*aOR* adjusted odds ratio, *CrI* credible interval, *Pr* probability, *P25* 25th percentile, *P50* 50th percentile, *P75* 75th percentile

aORs were estimated with potential confounders including maternal age, primiparity, maternal allergy history, smoking during pregnancy, pet ownership during pregnancy, sex of child, feeding at 6 months, nursery school attendance, maternal education level, annual family income and place of recruitment

## Discussion

### Main findings

The results relating to RQ2 are the primary finding of this study. A logistic regression analysis showed that physician-diagnosed asthma, ISAAC-based wheeze and ISAAC-based eczema in 12-month-old children were each associated with an initial inoculation in children aged less than 6 months of multiple types of inactivated vaccine as compared with an initial inoculation of a single type of inactivated vaccine. By using another statistical analysis, i.e. Bayesian multivariable logistic regression, the posterior probabilities of aOR > 1.0, aOR > 1.5 and even aOR > 2.0 confirmed the association of asthma with initial inoculations of four or five types of inactivated vaccines to be significantly high. It is possible that higher cumulative adjuvant exposures from initial immunisation with greater numbers of inactivated vaccines might influence an individual’s immunological status and lead to the development of a later allergic response.

### Inactivated vaccines

Related results have been reported by several other investigators. Kiraly et al. showed that delayed DTaP vaccination was associated with reduced eczema in Australia [5], and McDonald et al. found that delayed administration of the first dose of whole-cell DPT immunisation in childhood was negatively associated with the development of asthma [22]. In contrast, studies by Spycher et al. and by Vogt et al. each reported that routine immunisation against pertussis in infancy did not increase the risk of asthma or wheeze [7, 8]. Notably, these studies did not evaluate all inactivated vaccines but instead focused on only DPT, and their results were inconsistent. Here, we focused on initial vaccination with any type of inactivated vaccine during infancy to obtain the results presented above. Another report also examined the effect of multiple vaccinations on allergic diseases, but their results were different: Schlaud et al. reported that children with sufficient TDPHiHeP (tetanus, diphtheria, poliomyelitis, Hib, hepatitis B and pertussis) vaccination by the end of the first year of life had a lower risk of later developing hay fever [6]. Unlike the present work, that study included not only vaccines administered in the initial immunisation but also vaccines administered later during the first year of life. The discrepancy between their findings and our results may be due to the fact that the immune system develops and matures gradually over the course of infancy [23].

### More types of inactivated vaccines

Because inactivated vaccines generally contain adjuvants, our finding that initial immunisation with more types of inactivated vaccines is associated with the development of asthma or eczema at 12 months of age may implicate adjuvant exposure as a potential risk factor for the development of allergic diseases. We speculate that the mechanism for this association is the induction of type 2 immunological responses by adjuvants. However, not many cases of physician-diagnosed asthma are caused by type 2 immunity, including IgE-mediated allergic reactions at 12 months of age [24]. The present results regarding the effect of vaccination on physician-diagnosed asthma may be due to non-specific inflammation that is independent of type 2 immunity. In support of this possibility, Kashiwagi et al. reported that DPT/Hib/PCV immunisation increased the serum levels of a proinflammatory cytokine, IL-1β, compared with immunisation with DPT alone [25]. Although a weak association was found between eczema and initial immunisation with multiple inactive vaccines, further studies should be conducted to elucidate whether the effects of initial immunisation with multiple inactivated vaccines on asthma development persist after infancy, when most asthmatic children become type 2 immunity-dominant and IgE-dependent.

### BCG vaccination (live vaccine)

We did not generate any significant results in our comparison of initial immunisation with live versus inactivated vaccines for RQ1, suggesting that BCG vaccination may not have a preventive effect on the development of allergies. However, because BCG vaccination is known to promote Th1/Th17 immunity, we directly examined the effect of the initial immunisation being with BCG as RQ3 [9]. Our results failed to confirm the hypothesis that BCG vaccination of infants reduces the risk of development of allergic diseases. These findings agree with those of a systematic review and meta-analysis that concluded that BCG vaccine was not associated with the development of eczema, rhinoconjunctivitis, allergic sensitisation or allergies in general [10]. However, they differ from those of two other studies. Adults in Finland with a history of *Mycobacterium tuberculosis* (TB) infection prior to age 17 years had a lower prevalence of asthma than those without a prior TB infection [26]. In addition, Shirakawa et al. found that positive tuberculin responses were associated with a lower prevalence of asthma, a lower total IgE titre, lower Th2 cytokine (i.e. IL-4, IL-10 and IL-13) levels and a higher Th1 cytokine (IFN-γ) level in Japanese school children [27]. These two studies included adolescent and adult populations, whereas our study evaluated only young infants aged up to 1 year [26, 27]. The inconsistent results might stem from the different timings of outcome assessment between studies.

### Adjuvants

Adjuvants are essential to enhancing the adaptive immune response in the case of inactivated vaccines [28]. Aluminium is the most commonly used adjuvant in human inactivated vaccines because this adjuvant stimulates type 2 immune responses [2, 28]. Ohishi et al. found that the use of hydroxypropyl-β-cyclodextrin (HP-β-CD) as a vaccine adjuvant enhanced IgG antibody responses to a similar degree as the use of alum as an adjuvant but it induced fewer antigen-specific IgE responses compared with alum [29]. Although we were unable to identify the mechanism of how inactivated vaccines alter immunity because our study was epidemiological, we speculate that it involved the induction of type 2 responses. Additional studies are needed to confirm a causative link between adjuvants, type 2 immune responses and the development of allergic diseases. If this mechanism is confirmed, the development of alternate adjuvants that induce fewer type 2 responses might reduce the induction of allergy by initial immunisation with inactivated vaccines.

## Limitations

This study has several limitations. First, we could not confirm the information provided by the vaccine manufacturers, so we could not calculate an accurate cumulative dose of adjuvant based on the vaccine type. Notably, the Hib vaccine used in Japan (ActHIB®) does not include an adjuvant despite being an inactivated vaccine. However, because our RQs were based on distinguishing between live and inactivated vaccines, which we could do from the questionnaires, we were still able to complete our study, which focused on vaccination rather than adjuvant exposure. Second, the relatively large amount of missing data in our study resulted in a response bias. To address this limitation, we applied multiple imputation to account for missing values. We found no differences in the results between the complete case data and the multiple imputation data. Third, recall bias is a major concern in birth cohorts with self-reported questionnaires, which means that the prevalence of allergic diseases may have been underestimated. In addition, it was sometimes unclear at what ages in months the infants received their initial immunisation and developed allergic symptoms. The exact cause-and-effect relationship between exposures and outcomes was unclear. Notably, this study was unable to assess if children had allergic features during the first few months of life prior to vaccination. Because of this limitation, it is not possible to determine whether the reported allergies developed as a result of the effects of vaccination, so this study can evaluate only associations. Furthermore, the ISAAC questionnaire is not officially meant to be used for 1-year-old infants. However, various epidemiological studies have applied the ISAAC questionnaire for a wide range of ages [30]. Fourth, it was not possible to include children who had no clear history of immunisation in our comparisons. Fifth, we did not include all potential confounders, such as hospital visit frequency, in our analyses. The strength of this study is that it was conducted using a huge sample recruited from the general population across Japan in a nationwide prospective birth cohort study. Further studies are needed to validate our results.

## Conclusions

Although we recognise that immunisation is indispensable for the global elimination of various diseases, this large-scale birth cohort study demonstrated that the prevalence of asthma, wheeze and eczema in children at 12 months of age was associated with the administration of a larger number of types of inactivated vaccines at the initial immunisation before 6 months of age. Despite this association, we strongly support the global vaccination strategy and do not recommend that immunisation be halted. The results of this research support the reconsideration of better vaccination development in the future. Nonetheless, we highly encourage immunisation and support its current global strategy. The present results should be cautiously interpreted, but they suggest that the development of better vaccines and/or adjuvants could be beneficial.

## Supplementary information

**Additional file 1:.** Definitions of outcomes

**Additional file 2: Figure S1**. Flowchart of participant selection in this study

**Additional file 3: Table S1**. Summary of participant baseline characteristics. All data are presented as numbers (percentages). CQ: clinical question. **Table S2**. Summary of initial live vaccines and inactivated vaccines, RQ1. **Table S3**. Summary of the number of initial inactivated vaccines, RQ2. **Table S4**. Summary of number of initial BCG-only and rotavirus-only vaccines, RQ3

**Additional file 4: Table S5**. Prevalence for RQ1

**Additional file 5: Table S6**. Prevalence for RQ2

**Additional file 6: Table S7**. Prevalence for RQ3

## Data Availability

The data used to derive our conclusions are unsuitable for public deposition due to ethical restrictions and specific legal framework in Japan. It is prohibited by the Act on the Protection of Personal Information (Act No. 57 of 30 May 2003, amended on 9 September 2015) to publicly deposit data containing personal information. The Ethical Guidelines for Epidemiological Research enforced by the Japan Ministry of Education, Culture, Sports, Science and Technology and the Ministry of Health, Labour and Welfare also restrict the open sharing of the epidemiologic data. All inquiries about access to data should be sent to jecs-en@nies.go.jp. The person responsible for handling inquiries sent to this e-mail address is Dr Shoji F. Nakayama, JECS Programme Office, National Institute for Environmental Studies.

## Abbreviations

aOR: Adjusted odds ratio
BCG: Bacillus Calmette–Guérin
CI: Confidence interval
CQ: Clinical question
CrI: Credible interval
DTaP: Diphtheria, tetanus and acellular pertussis
Hib: *Haemophilus influenza* type b
IL: Interleukin
JECS: Japan Environment and Children’s Study
ISAAC: The International Study of Asthma and Allergies in Childhood
OR: Odds ratio
OTC: Over-the-counter
PCV: Pneumococcal conjugate *vaccine*
Pr: Probability
RCT: *Randomised controlled trial*
Th: T helper
UNICEF: United Nations Children’s Fund
WHO: World Health Organization
