# Supplementary material for: Cumulative inactivated vaccine exposure and allergy development among children: a birth cohort from Japan
Source: Environ Health Prev Med. 2020 Jul 7;25:27. doi: 10.1186/s12199-020-00864-7 (PMC7341599; doi:10.1186/s12199-020-00864-7)
Supplement: Supplementary file 1 — Additional file 1:. Definitions of outcomes [file 12199_2020_864_MOESM1_ESM.docx]

| **File S1**  Definitions of outcomes | | |
| --- | --- | --- |
| Outcomes of children at five years old | | |
| ISAAC-based wheeze | A positive answer to the question at 12 months of the children: “Has your child ever had wheezing or whistling in the chest at any time in the past?” (ISAAC) |  |
| ISAAC-based eczema | A positive answer to the question at 12 months of the children: “Has your child ever had itchy rash at any time in the past??” (ISAAC) |  |
| Physician-based asthma | A positive answer to the question at 12 months of the children: “Has your child ever been diagnosed with asthma by a physician?” |  |
| Physician-based atopic dermatitis | A positive answer to the question at 12 months of the children: “Has your child ever been diagnosed with atopic dermatitis by a physician?” |  |
| Physician-based food allergy | A positive answer to the question at 12 months of the children: “Has your child ever been diagnosed with food allergy by a physician?” |  |
| Allergy | A positive answer to any questions about eczema, asthma, atopic dermatitis and/or food allergy at 12 months of the children. |  |
