# Supplementary material for: Cumulative inactivated vaccine exposure and allergy development among children: a birth cohort from Japan
Source: Environ Health Prev Med. 2020 Jul 7;25:27. doi: 10.1186/s12199-020-00864-7 (PMC7341599; doi:10.1186/s12199-020-00864-7)
Supplement: Supplementary file 2 — Additional file 2: Figure S1. Flowchart of participant selection in this study [file 12199_2020_864_MOESM2_ESM.pptx]

## Slide 1
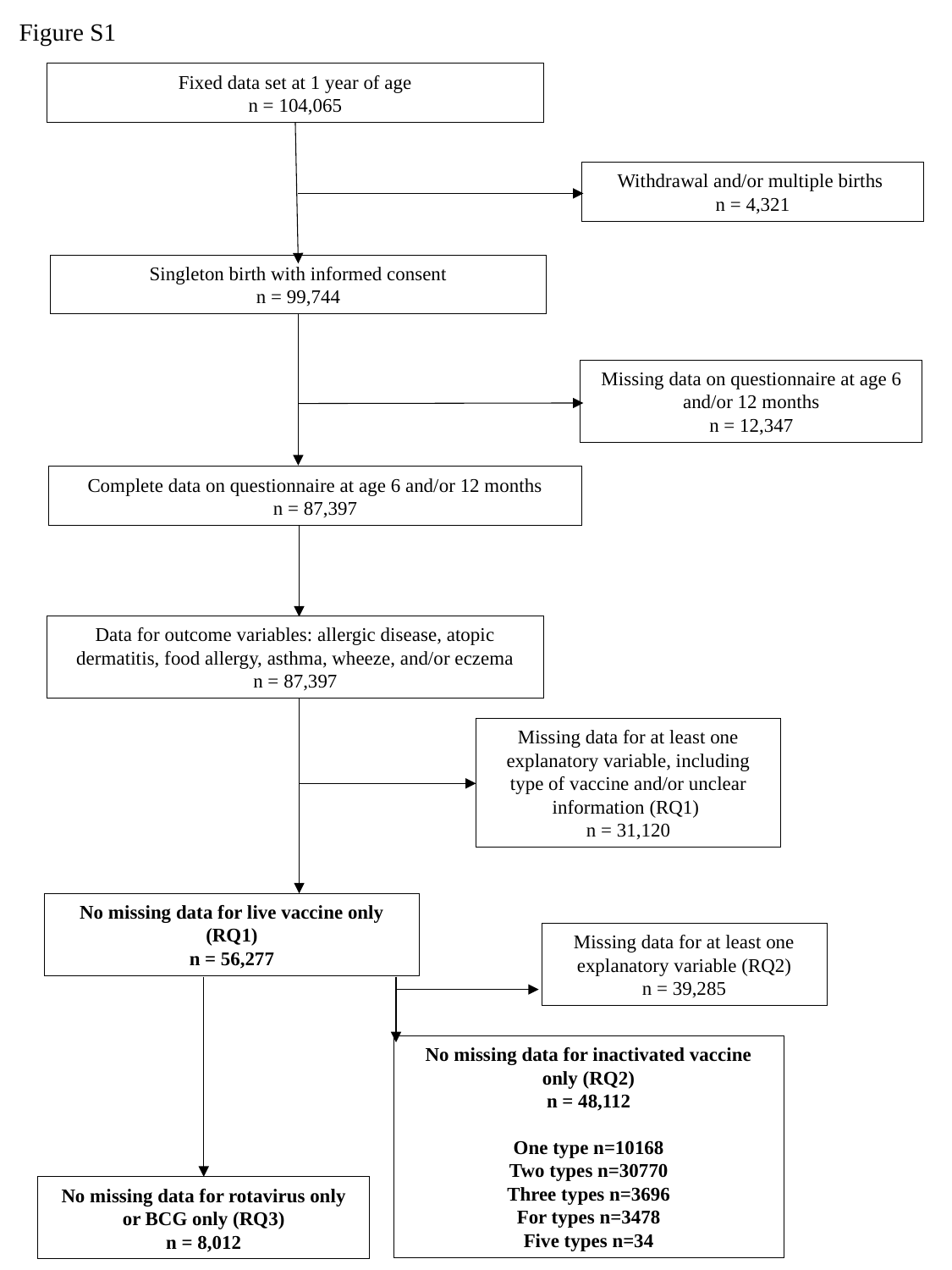

Figure S1
Fixed data set at 1 year of age
n = 104,065
Complete data on questionnaire at age 6 and/or 12 months
n = 87,397
No missing data for inactivated vaccine only (RQ2)
n = 48,112
One type n=10168
Two types n=30770
Three types n=3696
For types n=3478
Five types n=34
Missing data on questionnaire at age 6 and/or 12 months
n = 12,347
Missing data for at least one explanatory variable (RQ2)
n = 39,285
Withdrawal and/or multiple births
n = 4,321
Singleton birth with informed consent
n = 99,744
Data for outcome variables: allergic disease, atopic dermatitis, food allergy, asthma, wheeze, and/or eczema
n = 87,397
Missing data for at least one explanatory variable, including type of vaccine and/or unclear information (RQ1)
n = 31,120
No missing data for live vaccine only (RQ1)
n = 56,277
No missing data for rotavirus only or BCG only (RQ3)
n = 8,012
