# Supplementary material for: Cumulative inactivated vaccine exposure and allergy development among children: a birth cohort from Japan
Source: Environ Health Prev Med. 2020 Jul 7;25:27. doi: 10.1186/s12199-020-00864-7 (PMC7341599; doi:10.1186/s12199-020-00864-7)
Supplement: Supplementary file 3 — Additional file 3: Table S1. Summary of participant baseline characteristics. All data are presented as numbers (percentages). CQ: clinical question. Table S2. Summary of initial live vaccines and inactivated vaccines, RQ1. Table S3. Summary of the number of initial inactivated vaccines, RQ2. Table S4. Summary of number of initial BCG-only and rotavirus-only vaccines, RQ3 [file 12199_2020_864_MOESM3_ESM.doc]

**Table S1. Summary of participant baseline characteristics**

| **Variable** | **Category** | **RQ1**  **N=56277** | **RQ2**  **N=48112** | **RQ3**  **N=8012** |
| --- | --- | --- | --- | --- |
| Infant’s sex | Female | 27410 (48.7%) | 23488 (48.8%) | 3851 (48.1%) |
|  | Missing | 12 (0.0%) | 9 (0.0%) | 3 (0.0%) |
| Mother’s age at pregnancy | < 20 years | 501 (0.9%) | 414 (0.9%) | 85 (1.1%) |
|  | ≥ 20 & < 35 years | 41897 (74.4%) | 35883 (74.6%) | 5899 (73.6%) |
|  | ≥ 35 years | 13589 (24.1%) | 11560 (24.0%) | 1993 (24.9%) |
|  | Missing | 290 (0.5%) | 255 (0.5%) | 35 (0.4%) |
| Place of recruitment | Hokkaido | 4249 (7.6%) | 3798 (7.9%) | 448 (5.6%) |
|  | Fukushima/Miyagi | 11847 (21.1%) | 10235 (21.3%) | 1586 (19.8%) |
|  | Chiba/Kanagawa | 6390 (11.4%) | 5643 (11.7%) | 731 (9.1%) |
|  | Yamanashi | 3883 (6.9%) | 3176 (6.6%) | 686 (8.6%) |
|  | Toyama | 2824 (5.0%) | 2443 (5.1%) | 365 (4.6%) |
|  | Aichi | 2466 (4.4%) | 2003 (4.2%) | 451 (5.6%) |
|  | Kyoto/Osaka/Hyogo | 9606 (17.1%) | 7561 (15.7%) | 2013 (25.1%) |
|  | Tottori | 1869 (3.3%) | 1624 (3.4%) | 242 (3.0%) |
|  | Kochi | 4787 (8.5%) | 4333 (9.0%) | 446 (5.6%) |
|  | Fukuoka | 7795 (13.9%) | 6815 (14.2%) | 969 (12.1%) |
|  | Kumamoto/Miyazaki/Okinawa | 561 (1.0%) | 481 (1.0%) | 75 (0.9%) |
|  | Missing | 0 (0.0%) | 0 (0.0%) | 0 (0.0%) |
| Primiparous | Yes | 34562 (61.4%) | 29159 (60.6%) | 5297 (66.1%) |
|  | Missing | 310 (0.6%) | 269 (0.6%) | 41 (0.5%) |
| Maternal allergies | Yes | 28319 (50.6%) | 24242 (50.6%) | 3992 (50.0%) |
|  | Missing | 283 (0.5%) | 249 (0.5%) | 34 (0.4%) |
| Smoking during pregnancy | Yes | 2482 (4.5%) | 2092 (4.4%) | 385 (4.9%) |
|  | Missing | 644 (1.1%) | 563 (1.2%) | 81 (1.0%) |
| Pet ownership during pregnancy | Yes | 12792 (23.0%) | 10872 (22.9%) | 1886 (23.8%) |
|  | Missing | 615 (1.1%) | 535 (1.1%) | 79 (1.0%) |
| Education level | Junior high school | 2454 (4.4%) | 2077 (4.3%) | 366 (4.6%) |
|  | High school | 41699 (74.1%) | 35628 (74.1%) | 5958 (74.4%) |
|  | University | 11478 (20.4%) | 9851 (20.5%) | 1599 (20.0%) |
|  | Missing | 646 (1.1%) | 556 (1.2%) | 89 (1.1%) |
| Annual family income | > 6 million JPY | 12869 (24.7%) | 10979 (24.6%) | 1860 (25.3%) |
|  | Missing | 4085 (7.3%) | 3406 (7.1%) | 668 (8.3%) |
| Nutrition at 6 months | Exclusive breast feeding | 30940 (55.0%) | 26502 (55.1%) | 4358 (54.4%) |
|  | Missing | 2208 (3.9%) | 1809 (3.8%) | 392 (4.9%) |
| Nursery school at 6 months | Yes | 15164 (27.1%) | 13093 (27.3%) | 2031 (25.4%) |
|  | Missing | 228 (0.4%) | 196 (0.4%) | 31 (0.4%) |
| Allergic diseases | Yes | 17346 (30.9%) | 14805 (30.9%) | 2495 (31.3%) |
|  | Missing | 211 (0.4%) | 179 (0.4%) | 32 (0.4%) |
| Atopic dermatitis | Yes | 2449 (4.4%) | 2065 (4.3%) | 374 (4.7%) |
|  | Missing | 0 (0.0%) | 0 (0.0%) | 0 (0.0%) |
| Food allergy | Yes | 9496 (16.9%) | 8126 (16.9%) | 1348 (16.8%) |
|  | Missing | 0 (0.0%) | 0 (0.0%) | 0 (0.0%) |
| Asthma | Yes | 1466 (2.6%) | 1258 (2.6%) | 204 (2.5%) |
|  | Missing | 0 (0.0%) | 0 (0.0%) | 0 (0.0%) |
| Wheezing | Yes | 11166 (19.9%) | 9547 (19.9%) | 1584 (19.9%) |
|  | Missing | 286 (0.5%) | 247 (0.5%) | 38 (0.5%) |
| Eczema | Yes | 10319 (18.4%) | 8799 (18.4%) | 1487 (18.7%) |
|  | Missing | 266 (0.5%) | 226 (0.5%) | 40 (0.5%) |

All data are presented as numbers (percentages).

CQ: clinical question.

**Table S2. Summary of initial live vaccines and inactivated vaccines, RQ1**

| **Arm** | **Pattern** | **Combination** | **N (%)** |
| --- | --- | --- | --- |
| Live | All |  | 8165 (14.5%) |
|  | One type | BCG | 6093 (10.8%) |
|  |  | Poliomyelitis (Live) | 45 (0.1%) |
|  |  | Rota | 1919 (3.4%) |
|  | Two types | BCG+Poliomyelitis (Live) | 57 (0.1%) |
|  |  | BCG+Rota | 51 (0.1%) |
|  |  | Poliomyelitis (Live)+Rota | 0 (0.0%) |
|  | Three types | BCG+Poliomyelitis (Live)+Rota | 0 (0.0%) |
| Inactivated | All |  | 48112 (85.5%) |
|  | One type | DPT | 853 (1.5%) |
|  |  | Poliomyelitis (Inactivated) | 58 (0.1%) |
|  |  | Influenza | 28 (0.0%) |
|  |  | Hib | 7314 (13.0%) |
|  |  | Pneumococcus | 1692 (3.0%) |
|  |  | Hepatitis B | 223 (0.4%) |
|  | Two types | DPT+Poliomyelitis (Inactivated) | 4076 (7.2%) |
|  |  | DPT+Influenza | 87 (0.2%) |
|  |  | DPT+Hib | 6328 (11.2%) |
|  |  | DPT+Pneumococcus | 6041 (10.7%) |
|  |  | DPT+Hepatitis B | 225 (0.4%) |
|  |  | Poliomyelitis (Inactivated)+Influenza | 48 (0.1%) |
|  |  | Poliomyelitis (Inactivated)+Hib | 3631 (6.5%) |
|  |  | Poliomyelitis (Inactivated)+Pneumococcus | 3501 (6.2%) |
|  |  | Poliomyelitis (Inactivated)+Hepatitis B | 76 (0.1%) |
|  |  | Influenza+Hib | 99 (0.2%) |
|  |  | Influenza+Pneumococcus | 249 (0.4%) |
|  |  | Influenza+Hepatitis B | 21 (0.0%) |
|  |  | Hib+Pneumococcus | 36368 (64.6%) |
|  |  | Hib+Hepatitis B | 1075 (1.9%) |
|  |  | Pneumococcus+Hepatitis B | 1037 (1.8%) |
|  | Three types | DPT+Poliomyelitis (Inactivated)+Influenza | 39 (0.1%) |
|  |  | DPT+Poliomyelitis (Inactivated)+Hib | 3429 (6.1%) |
|  |  | DPT+Poliomyelitis (Inactivated)+Pneumococcus | 3310 (5.9%) |
|  |  | DPT+Poliomyelitis (Inactivated)+Hepatitis B | 57 (0.1%) |
|  |  | DPT+Influenza+Hib | 32 (0.1%) |
|  |  | DPT+Influenza+Pneumococcus | 65 (0.1%) |
|  |  | DPT+Influenza+Hepatitis B | 6 (0.0%) |
|  |  | DPT+Hib+Pneumococcus | 5846 (10.4%) |
|  |  | DPT+Hib+Hepatitis B | 203 (0.4%) |
|  |  | DPT+Pneumococcus+Hepatitis B | 202 (0.4%) |
|  |  | Poliomyelitis (Inactivated)+Influenza+Hib | 17 (0.0%) |
|  |  | Poliomyelitis (Inactivated)+Influenza+Pneumococcus | 34 (0.1%) |
|  |  | Poliomyelitis (Inactivated)+Influenza+Hepatitis B | 2 (0.0%) |
|  |  | Poliomyelitis (Inactivated)+Hib+Pneumococcus | 3382 (6.0%) |
|  |  | Poliomyelitis (Inactivated)+Hib+Hepatitis B | 61 (0.1%) |
|  |  | Poliomyelitis (Inactivated)+Pneumococcus+Hepatitis B | 59 (0.1%) |
|  |  | Influenza+Hib+Pneumococcus | 49 (0.1%) |
|  |  | Influenza+Hib+Hepatitis B | 6 (0.0%) |
|  |  | Influenza+Pneumococcus+Hepatitis B | 16 (0.0%) |
|  |  | Hib+Pneumococcus+Hepatitis B | 997 (1.8%) |
|  | Four types | DPT+Poliomyelitis (Inactivated)+Influenza+Hib | 10 (0.0%) |
|  |  | DPT+Poliomyelitis (Inactivated)+Influenza+Pneumococcus | 28 (0.0%) |
|  |  | DPT+Poliomyelitis (Inactivated)+Influenza+Hepatitis B | 2 (0.0%) |
|  |  | DPT+Poliomyelitis (Inactivated)+Hib+Pneumococcus | 3223 (5.7%) |
|  |  | DPT+Poliomyelitis (Inactivated)+Hib+Hepatitis B | 43 (0.1%) |
|  |  | DPT+Poliomyelitis (Inactivated)+Pneumococcus+Hepatitis B | 42 (0.1%) |
|  |  | DPT+Influenza+Hib+Pneumococcus | 15 (0.0%) |
|  |  | DPT+Influenza+Hib+Hepatitis B | 1 (0.0%) |
|  |  | DPT+Influenza+Pneumococcus+Hepatitis B | 5 (0.0%) |
|  |  | DPT+Hib+Pneumococcus+Hepatitis B | 186 (0.3%) |
|  |  | Poliomyelitis (Inactivated)+Influenza+Hib+Pneumococcus | 6 (0.0%) |
|  |  | Poliomyelitis (Inactivated)+Influenza+Hib+Hepatitis B | 0 (0.0%) |
|  |  | Poliomyelitis (Inactivated)+Influenza+Pneumococcus+Hepatitis B | 2 (0.0%) |
|  |  | Poliomyelitis (Inactivated)+Hib+Pneumococcus+Hepatitis B | 48 (0.1%) |
|  |  | Influenza+Hib+Pneumococcus+Hepatitis B | 3 (0.0%) |
|  | Five types | DPT+Poliomyelitis (Inactivated)+Influenza+Hib+Pneumococcus | 0 (0.0%) |
|  |  | DPT+Poliomyelitis (Inactivated)+Influenza+Hib+Hepatitis B | 0 (0.0%) |
|  |  | DPT+Poliomyelitis (Inactivated)+Influenza+Pneumococcus+Hepatitis B | 2 (0.0%) |
|  |  | DPT+Poliomyelitis (Inactivated)+Hib+Pneumococcus+Hepatitis B | 32 (0.1%) |
|  |  | DPT+Influenza+Hib+Pneumococcus+Hepatitis B | 0 (0.0%) |
|  |  | Poliomyelitis (Inactivated)+Influenza+Hib+Pneumococcus+Hepatitis B d | 0 (0.0%) |

**Table S3. Summary of the number of initial inactivated vaccines, RQ2**

| **Arm** | **Pattern** | **Combination** | **N (%)** |
| --- | --- | --- | --- |
|  | All | - | 48112 (100.0%) |
| Inactivated 1 | One type |  | 10168 (21.1%) |
|  |  | DPT | 853 (1.8%) |
|  |  | Poliomyelitis (Inactivated) | 58 (0.1%) |
|  |  | Influenza | 28 (0.1%) |
|  |  | Hib | 7314 (15.2%) |
|  |  | Pneumococcus | 1692 (3.5%) |
|  |  | Hepatitis B | 223 (0.5%) |
| Inactivated 2 | Two types |  | 30770 (64.0%) |
|  |  | DPT+Poliomyelitis (Inactivated) | 4076 (8.5%) |
|  |  | DPT+Influenza | 87 (0.2%) |
|  |  | DPT+Hib | 6328 (13.2%) |
|  |  | DPT+Pneumococcus | 6041 (12.6%) |
|  |  | DPT+Hepatitis B | 225 (0.5%) |
|  |  | Poliomyelitis (Inactivated)+Influenza | 48 (0.1%) |
|  |  | Poliomyelitis (Inactivated)+Hib | 3631 (7.5%) |
|  |  | Poliomyelitis (Inactivated)+Pneumococcus | 3501 (7.3%) |
|  |  | Poliomyelitis (Inactivated)+Hepatitis B | 76 (0.2%) |
|  |  | Influenza+Hib | 99 (0.2%) |
|  |  | Influenza+Pneumococcus | 249 (0.5%) |
|  |  | Influenza+Hepatitis B | 21 (0.0%) |
|  |  | Hib+Pneumococcus | 36368 (75.6%) |
|  |  | Hib+Hepatitis B | 1075 (2.2%) |
|  |  | Pneumococcus+Hepatitis B | 1037 (2.2%) |
| Inactivated 3 | Three types |  | 3696 (7.7%) |
|  |  | DPT+Poliomyelitis (Inactivated)+Influenza | 39 (0.1%) |
|  |  | DPT+Poliomyelitis (Inactivated)+Hib | 3429 (7.1%) |
|  |  | DPT+Poliomyelitis (Inactivated)+Pneumococcus | 3310 (6.9%) |
|  |  | DPT+Poliomyelitis (Inactivated)+Hepatitis B | 57 (0.1%) |
|  |  | DPT+Influenza+Hib | 32 (0.1%) |
|  |  | DPT+Influenza+Pneumococcus | 65 (0.1%) |
|  |  | DPT+Influenza+Hepatitis B | 6 (0.0%) |
|  |  | DPT+Hib+Pneumococcus | 5846 (12.2%) |
|  |  | DPT+Hib+Hepatitis B | 203 (0.4%) |
|  |  | DPT+Pneumococcus+Hepatitis B | 202 (0.4%) |
|  |  | Poliomyelitis (Inactivated)+Influenza+Hib | 17 (0.0%) |
|  |  | Poliomyelitis (Inactivated)+Influenza+Pneumococcus | 34 (0.1%) |
|  |  | Poliomyelitis (Inactivated)+Influenza+Hepatitis B | 2 (0.0%) |
|  |  | Poliomyelitis (Inactivated)+Hib+Pneumococcus | 3382 (7.0%) |
|  |  | Poliomyelitis (Inactivated)+Hib+Hepatitis B | 61 (0.1%) |
|  |  | Poliomyelitis (Inactivated)+Pneumococcus+Hepatitis B | 59 (0.1%) |
|  |  | Influenza+Hib+Pneumococcus | 49 (0.1%) |
|  |  | Influenza+Hib+Hepatitis B | 6 (0.0%) |
|  |  | Influenza+Pneumococcus+Hepatitis B | 16 (0.0%) |
|  |  | Hib+Pneumococcus+Hepatitis B | 997 (2.1%) |
| Inactivated 4 & 5 | Four types |  | 3478 (7.2%) |
|  |  | DPT+Poliomyelitis (Inactivated)+Influenza+Hib | 10 (0.0%) |
|  |  | DPT+Poliomyelitis (Inactivated)+Influenza+Pneumococcus | 28 (0.1%) |
|  |  | DPT+Poliomyelitis (Inactivated)+Influenza+Hepatitis B | 2 (0.0%) |
|  |  | DPT+Poliomyelitis (Inactivated)+Hib+Pneumococcus | 3223 (6.7%) |
|  |  | DPT+Poliomyelitis (Inactivated)+Hib+Hepatitis B | 43 (0.1%) |
|  |  | DPT+Poliomyelitis (Inactivated)+Pneumococcus+Hepatitis B | 42 (0.1%) |
|  |  | DPT+Influenza+Hib+Pneumococcus | 15 (0.0%) |
|  |  | DPT+Influenza+Hib+Hepatitis B | 1 (0.0%) |
|  |  | DPT+Influenza+Pneumococcus+Hepatitis B | 5 (0.0%) |
|  |  | DPT+Hib+Pneumococcus+Hepatitis B | 186 (0.4%) |
|  |  | Poliomyelitis (Inactivated)+Influenza+Hib+Pneumococcus | 6 (0.0%) |
|  |  | Poliomyelitis (Inactivated)+Influenza+Hib+Hepatitis B | 0 (0.0%) |
|  |  | Poliomyelitis (Inactivated)+Influenza+Pneumococcus+Hepatitis B | 2 (0.0%) |
|  |  | Poliomyelitis (Inactivated)+Hib+Pneumococcus+Hepatitis B | 48 (0.1%) |
|  |  | Influenza+Hib+Pneumococcus+Hepatitis B | 3 (0.0%) |
|  | Five types | DPT+Poliomyelitis (Inactivated)+Influenza+Hib+Pneumococcus | 0 (0.0%) |
|  |  | DPT+Poliomyelitis (Inactivated)+Influenza+Hib+Hepatitis B | 0 (0.0%) |
|  |  | DPT+Poliomyelitis (Inactivated)+Influenza+Pneumococcus+Hepatitis B | 2 (0.0%) |
|  |  | DPT+Poliomyelitis (Inactivated)+Hib+Pneumococcus+Hepatitis B | 32 (0.1%) |
|  |  | DPT+Influenza+Hib+Pneumococcus+Hepatitis B | 0 (0.0%) |
|  |  | Poliomyelitis (Inactivated)+Influenza+Hib+Pneumococcus+Hepatitis B | 0 (0.0%) |

**Table S4. Summary of number of initial BCG-only and rotavirus-only vaccines, RQ3**

| **Arm** |  | **N (%)** |
| --- | --- | --- |
| Live vaccine arm (one type) | BCG | 6093 (76.0%) |
| Live vaccine arm (one type) | Rotavirus | 1919 (24.0%) |
