# Supplementary material for: Cumulative inactivated vaccine exposure and allergy development among children: a birth cohort from Japan
Source: Environ Health Prev Med. 2020 Jul 7;25:27. doi: 10.1186/s12199-020-00864-7 (PMC7341599; doi:10.1186/s12199-020-00864-7)
Supplement: Supplementary file 4 — Additional file 4: Table S5. Prevalence for RQ1 [file 12199_2020_864_MOESM4_ESM.rtf]

Table S5 Prevalence for RQ1


Prevalence of Allergy by Vaccine types
Group Category	Rate	
Live	2541/8165 (31.1%)	
Inactivate	14805/48112 (30.8%)	


Prevalence of Atopic Disease by Vaccine types
Group Category	Rate	
Live	384/8165 (4.7%)	
Inactivate	2065/48112 (4.3%)	


Prevalence of Food Allergy by Vaccine types
Group Category	Rate	
Live	1370/8165 (16.8%)	
Inactivate	8126/48112 (16.9%)	


Prevalence of Asthma by Vaccine types
Group Category	Rate	
Live	208/8165 (2.5%)	
Inactivate	1258/48112 (2.6%)	


Prevalence of Wheezing by Vaccine types
Group Category	Rate	
Live	1619/8165 (19.8%)	
Inactivate	9547/48112 (19.8%)	


Prevalence of Eczema by Vaccine types
Group Category	Rate	
Live	1520/8165 (18.6%)	
Inactivate	8799/48112 (18.3%)	
