# Supplementary material for: Cumulative inactivated vaccine exposure and allergy development among children: a birth cohort from Japan
Source: Environ Health Prev Med. 2020 Jul 7;25:27. doi: 10.1186/s12199-020-00864-7 (PMC7341599; doi:10.1186/s12199-020-00864-7)
Supplement: Supplementary file 5 — Additional file 5: Table S6. Prevalence for RQ2 [file 12199_2020_864_MOESM5_ESM.rtf]

Table S6 Prevalence for RQ2

Prevalence of Allergy by Number of Inactivate Vaccines
Group Category	Rate	
1	3099/10168 (30.5%)	
2	9449/30770 (30.7%)	
3	1147/3696 (31.0%)	
4 or 5	1110/3478 (31.9%)	

Prevalence of Atopic Disease by Number of Inactivate Vaccines
Group Category	Rate	
1	441/10168 (4.3%)	
2	1279/30770 (4.2%)	
3	178/3696 (4.8%)	
4 or 5	167/3478 (4.8%)	

Prevalence of Food Allergy by Number of Inactivate Vaccines
Group Category	Rate	
1	1798/10168 (17.7%)	
2	5166/30770 (16.8%)	
3	591/3696 (16.0%)	
4 or 5	571/3478 (16.4%)	

Prevalence of Asthma by Number of Inactivate Vaccines
Group Category	Rate	
1	208/10168 (2.0%)	
2	800/30770 (2.6%)	
3	124/3696 (3.4%)	
4 or 5	126/3478 (3.6%)	

Prevalence of Wheezing by Number of Inactivate Vaccines
Group Category	Rate	
1	1924/10168 (18.9%)	
2	5988/30770 (19.5%)	
3	853/3696 (23.1%)	
4 or 5	782/3478 (22.5%)	

Prevalence of Eczema by Number of Inactivate Vaccines
Group Category	Rate	
1	1818/10168 (17.9%)	
2	5599/30770 (18.2%)	
3	704/3696 (19.1%)	
4 or 5	678/3478 (19.5%)	
