# Supplementary material for: Cumulative inactivated vaccine exposure and allergy development among children: a birth cohort from Japan
Source: Environ Health Prev Med. 2020 Jul 7;25:27. doi: 10.1186/s12199-020-00864-7 (PMC7341599; doi:10.1186/s12199-020-00864-7)
Supplement: Supplementary file 6 — Additional file 6: Table S7. Prevalence for RQ3 [file 12199_2020_864_MOESM6_ESM.rtf]

Table S7 Prevalence for RQ3

Prevalence of Allergy by Live Vaccine types
Group Category	Rate	
BCG	1888/6093 (31.0%)	
Rota	607/1919 (31.6%)	

Prevalence of Atopic Disease by Live Vaccine types
Group Category	Rate	
BCG	293/6093 (4.8%)	
Rota	81/1919 (4.2%)	

Prevalence of Food Allergy by Live Vaccine types
Group Category	Rate	
BCG	996/6093 (16.4%)	
Rota	352/1919 (18.3%)	

Prevalence of Asthma by Live Vaccine types
Group Category	Rate	
BCG	164/6093 (2.7%)	
Rota	40/1919 (2.1%)	

Prevalence of Wheezing by Live Vaccine types
Group Category	Rate	
BCG	1225/6093 (20.1%)	
Rota	359/1919 (18.7%)	

Prevalence of Eczema by Live Vaccine types
Group Category	Rate	
BCG	1134/6093 (18.6%)	
Rota	353/1919 (18.4%)	
